# Hand injuries in an older population - a retrospective cohort study from a single hand surgery centre

**DOI:** 10.1186/s12891-019-2617-x

**Published:** 2019-05-24

**Authors:** Olof Kringstad, Lars B. Dahlin, Hans-Eric Rosberg

**Affiliations:** 1 Department of Hand Surgery, Skåne University Hospital, Jan Waldenströms gata 5, SE-205 02 Malmö, Sweden; 2 Department of Translational Medicine - Hand Surgery, Lund University, Malmö, Sweden

**Keywords:** Hand injury, Elderly, Incidence, Injury severity, Comorbidity

## Abstract

**Background:**

Hand injuries occur at all ages. With an aging population globally an increasing number of hand injuries among the elderly is to be expected. The aim of the present study is to describe the health characteristics and detailed injury patterns for elderly with hand injuries, with incidence, as a background for further studies on the topic. Specific knowledge is currently lacking about hand injuries among this group. The study is a retrospective cohort study from a single hand surgery centre.

**Methods:**

Data were collected for 286 patients, aged > 65 years, treated for traumatic hand injury between July 1, 2013 and June 30, 2014 at the Department of Hand Surgery in Malmö.

**Results:**

Incidence was 21.3/10000 inhabitants/year. The 286 patients included comprised 145 women and 141 men. The men had more severe injuries, often involving a wound, while women most commonly sustained a fracture after a fall. The men were younger than the women and required more surgery/admissions. Among all patients, 13% were healthy, while 27% patients took ≥5 drugs, mainly for cardiovascular disease.

**Conclusions:**

The incidence of hand injuries among the elderly is lower than among a younger population. Men sustained more wounds from using hazardous equipment, while women sustained post-fall fractures. A minority of the elderly is healthy. Prevention of fall injuries is crucial and emphasising safety awareness might reduce injuries in both sexes.

## Background

Among the elderly, women injure their hands more often than men, commonly sustaining fractures due to falls [1]. Falling is the most common injury mechanism in people aged over 65 years, accounting for 75–79% of the traumas [2, 3]. Approximately one third of the over-65 population fall every year [4] and one sixth fall repeatedly [5]. Studies show that 14% of the injuries among the elderly seen at emergency departments are to the upper extremities [3], while 29% of all injuries presenting at emergency departments are to the hand and wrist [6]. With the mean age of populations increasing in the developed and developing world, the number and proportion of elderly people will also increase [7]. Hence, an increasing number of injuries among the elderly can be expected [2]. Information is needed of the specific pattern of hand injuries that affect elderly. However, there are no such studies on elderly, focusing only on specific hand injuries in general and with details about the patient population. Such information may be relevant when planning how the resources should be used in the health care system in relation to the present different living patterns among elderly, i.e. more active at older age, which is highlighted in population studies [8].

More than 50% of older trauma patients suffer from cardiovascular disease [2]. Other conditions, such as balance difficulties, visual impairment and reduced cognitive capacity, may increase the risk of trauma and complicate subsequent treatment. Older trauma patients may make higher demands on the healthcare system than younger patients, due to a combination of increasing rates of both co-morbidity and poly-pharmacy, as a result of wider use of cardio-protective medicines, for example [2, 4, 9].

Only one study has highlighted treatment of severe hand injuries among patients aged > 65 years, concluding that age is no contraindication regarding replantation of fingers [10]. Healthcare consumption for injuries among the elderly is high, giving rise to substantial costs. Preventive measures would be beneficial in reducing these costs, avoiding overburdening of the healthcare system and reducing morbidity in this population [4, 11–14].

Our aim is to retrospective describe the health characteristics and detailed injury patterns for elderly with hand injuries in a cohort study from a single hand surgery centre, with a calculated incidence, as a background for further studies on the topic, since there is a lack of knowledge about specific hand injuries among the elderly.

## Methods

### Subjects

We included retrospectively all patients, older than 65 years, with a hand injury defined according to ICD-10 codes, from July 1, 2013 to June 30, 2014, who were treated at the Department of Hand Surgery in Malmö. The department treats patients with both minor and major hand injuries from the cities of Malmö and Lund in agreement with the involved health care providers in the region. The patients from Malmö and Lund are referred from their family doctor, or from the department of Orthopaedics or from the A&E Department.

In 2013/14 the city of Malmö had a population of 426,849 of whom 66,736 were older than 65 years. Lund had a population of 220,979 with 35,343 people older than 65 years. The hand injury incidence will be calculated for both these two regions. The annual report (Statistics Sweden) for 2013 was used as December 31, 2013, which is the exact halfway point in the study period; hence provides an average statistical base to calculate the incidence.

Patients with major hand injuries, requiring specialized hand surgical care, including flexor tendon injuries and nerve injuries, are also referred to the department from the Southern healthcare region in Sweden with a population, in 2013/14, of 1,741,584 including 323,954 people aged over 65 years.

### Variables

Data were collected in a standardised manner for each patient from their medical records with the following variables: *deceased or not, gender, age when injured, previous hand injuries, hand dominance, time and date of injury, injured side.* Further data were also collected as follows.

### Co-morbidity

For each patient the number of diagnoses was counted and those without any, explicitly mentioned in the records, were labelled healthy. Examples of diagnoses that were categorized, relevant from a hand condition and injury perspective, were cardiovascular and metabolic diseases, osteoarthritis, dizziness, visual impairment, psychiatric disorders, and osteoporosis.

### Number of pharmaceutical drugs

The number of prescribed drugs was counted and the patients were divided into three groups 0, 1–4 and ≥ 5 pharmaceutical drugs administered.

### Site of injury

Sites of injury were grouped into carpal/forearm, rays 1 to 5 and multiple sites.

### Type of injury

The main types of injuries were grouped into three categories: fractures, wounds and dislocations/ligament injuries.

### Injured structure

Every injury was described according to the Modified Hand Injury Severity Score (MHISS) [15, 16].

### Injury severity

MHISS was also used to score the severity of the injuries and to group them into four hand-injury categories: Minor (< 20), Moderate (21–50), Severe (51–100) and Major (> 100) [15, 16].

### Settings for injuries

The settings in which the injuries took place were grouped into five categories: outdoors, home/indoors, when engaged in carpentry or handling firewood, at work, in/on a motor vehicle.

### Injury mechanism

The injury mechanisms were grouped into six categories: fall of any kind, cut or saw, crush/avulsion, pull/punch/twist, traffic accident and bite.

### Reason for injury

If specified, the causes of the injury were grouped into six categories: tripping/stumbling/slipping, power-tools, animal, door/heavy object, glass and other sharp objects and cutting tools.

### Additional injuries

Additional injuries that occurred in conjunction with the hand injuries were categorized into three main groups: wounds/contusions or fractures to the head, costal and vertebral fractures/compressions and lower-extremity fractures.

### Treatment information

Patients were categorized as being treated either as out-patients or in-patients. The type of treatment was recorded as either conservative (i.e. plaster or bandage) or operative (ranging from simple sutures to full scale surgery). The number of days spent on a hand surgical ward was counted for the in-patients and the number of visits to a hand surgeon, nurse and hand therapist for out-patients. Some patients with critical physical conditions were treated as in-patients on other wards; for these patients the hand surgical interventions were counted as out-patient visits due to the difficulty of determining how many in-patient days the hand injury alone would have required. Whether the eventual use of antibiotics was prophylactic or due to infection was noted. Complications comprised infection or other causes. The number of re-operations was grouped into 1, 2 or more.

### Statistical methods

Data are presented as a median [25th–75th percentiles] or numbers (%). The Mann-Whitney U test was used for continuous variables, Pearson’s Chi-square test and Fisher’s exact test (in small sample groups) were used for categorical variables. A *p*-value of 0.05 or less was considered significant.

## Results

### Incidence, gender, injured hand and time of injury

**Fig. 1.**
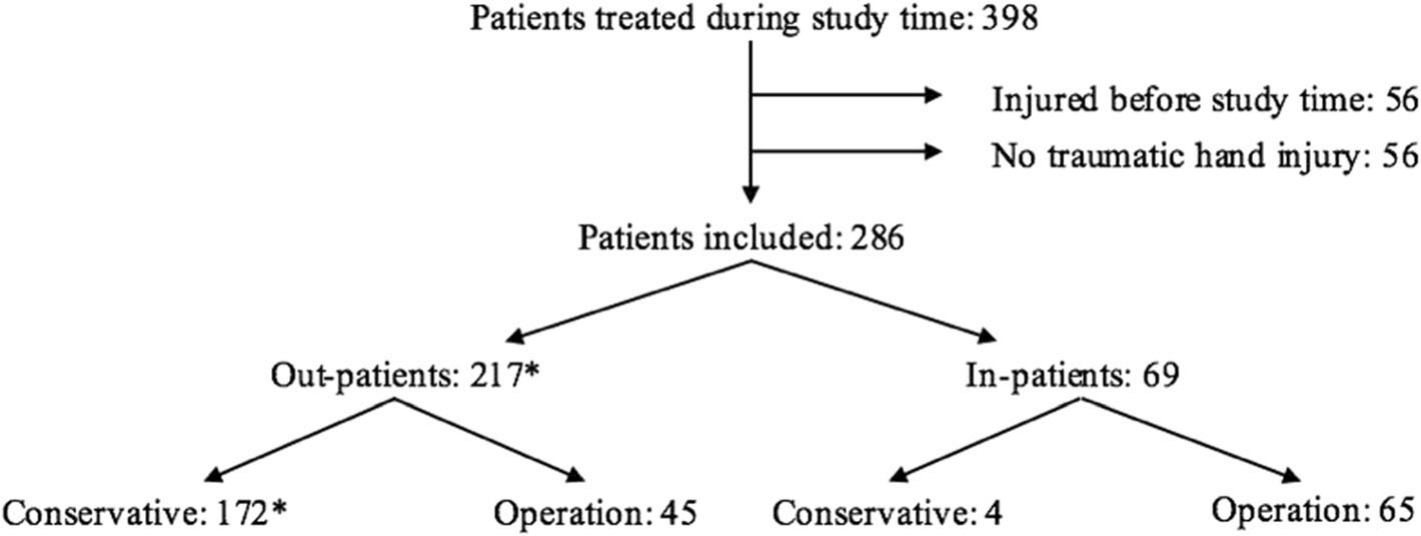
Inclusion process and the distribution between in- and out-patients and type of treatment. *One woman received conservative treatment twice for two different injuries

Three hundred and ninety-eight patients were treated during the period of the study. Fifty-six patients were injured before July 1, 2013; 40 patients received treatment for conditions other than a traumatic hand or forearm injury; and 16 patients had only contusions. Thus, the remaining 286 patients were included; 217/286 (76%) were treated as out-patients and 69/286 (24%) as in-patients (Fig. 1).

One woman sustained two injuries during the study period. In the subsequent calculations she is counted as one patient, but each injury is considered separately. Consequently, 287 injuries are included. Nine men and seven women were no longer alive when the data were collected.

The overall incidence of traumatic hand injury in the elderly was 21.3/10000 inhabitants per year in the Malmö and Lund regions combined. Table 1 presents the number and the incidence of traumatic hand injuries for men and women in these regions, divided into 5-year age groups, showing a slight reduction in incidence as age increases.

**Table 1:** The number of hand injuries and the incidence/10000 inhabitants and year

	Lund		Malmö		Total
Age (years)	Women	Men	Women	Men	
66–70	12 (18.7)	13 (21.8)	24 (21.8)	30 (29.2)	79 (23.4)
71–75	10 (21.6)	7 (16.7)	22 (26.1)	19 (25.6)	58 (23.3)
76–80	5 (15.6)	3 (11.0)	20 (30.1)	11 (21.3)	39 (22.0)
81–85	5 (20.0)	1 (5.7)	9 (16.5)	13 (36.6)	28 (21.1)
86–90	3 (18.4)	1 (10.6)	6 (15.4)	1 (5.3)	11 (13.2)
91–95	1 (12.1)	1 (33.6)	1 (5.2)	0	3 (8.1)
≥96	0	0	0	0	0
Total	36 (18.5)	26 (16.3)	82 (21.8)	74 (25.4)	218 (21.3)

Traumatic hand injuries occurred in equal proportions among elderly women and men in the two regions. More men than women were referred to the department from other regions; 28 women and 41 men (*p* = 0.02). Male patients were significantly younger than female (*p* < 0.01; Table 2). Handedness was registered for only one third, 96/286, of the patients (Table 2). The left hand was more commonly injured than the right among both men (*p* = 0.05) and women (*p* < 0.01).

**Table 2:** Characteristics for 286 patients with a hand injury treated at a department for hand surgery

Patient characteristics	Women *n* = 145 (51%)	Men *n* = 141 (49%)	Total *n* = 286	*P*-value
Age	74 [70–79]	71 [68–76]	72 [69–78]	^a^ **< 0.01**
Co-morbidity				
Healthy	17 (12%)	19 (13%)	36 (13%)	^**b**^0.65
Hypertension	57 (39%)	73 (52%)	130 (45%)	^**b**^ **0.03**
Cardiovascular disease	38 (26%)	58 (41%)	96 (34%)	^**b**^ **< 0.01**
Diabetes mellitus type 2	9 (6%)	26 (18%)	35 (12%)	^**b**^ **< 0.01**
Pulmonary disease	19 (13%)	15 (11%)	34 (12%)	^**b**^0.52
Thyroid disorders	25 (17%)	5 (4%)	30 (10%)	^**b**^ **< 0.01**
Osteoarthritis	21 (14%)	8 (6%)	29 (10%)	^**b**^ **0.01**
Number of medicines	2 [1–5]	3 [1–6]	2 [1–5]	^**c**^0.13
0	27 (19%)	23 (16%)	50 (17%)	^**b**^0.64
1–4	59 (42%)	54 (38%)	113 (40%)	^**b**^0.75
**≥**5 (5–17)	32 (22%)	46 (33%)	78 (27%)	^**b**^0.09
Missing data	27 (19%)	18 (13%)	45 (16%)	^**b**^0.17
Previous hand injury	10 (7%)	12 (9%)	22 (8%)	^b^0.61
Handedness				
Right	44 (30%)	49 (35%)	93 (33%)	^**b**^0.51
Left	2 (1%)	2 (1%)	4 (1%)	–
Bimanual	1 (1%)	0	1 (0%)	–
Missing	98 (68%)	90 (64%)	188 (66%)	^**b**^0.64

*Values are presented as number of patients (% of patient group) or median [25th – 75th percentiles]. P-values represent differences between men and women in number of patients affected or median values by the use of* ^*a*^ *Mann-Whitney U test for continuous variables and* ^*b*^ *Pearson’s Chi-square test or* ^*c*^ *Fisher’s exact test (in small sample groups) for categorical variables*

**Fig. 2:**
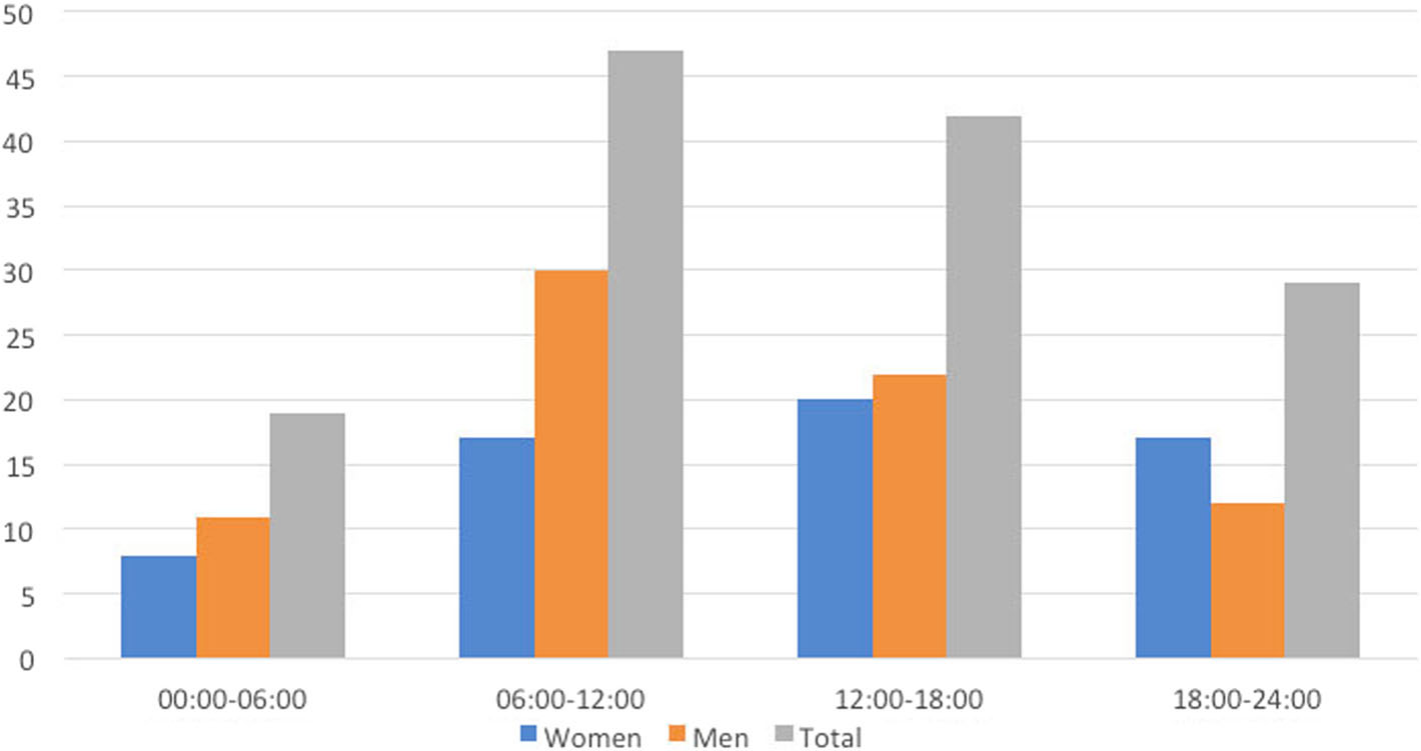
Number of injuries in each quarter of the day. Data missing for 84 women and 66 men

**Fig. 3:**
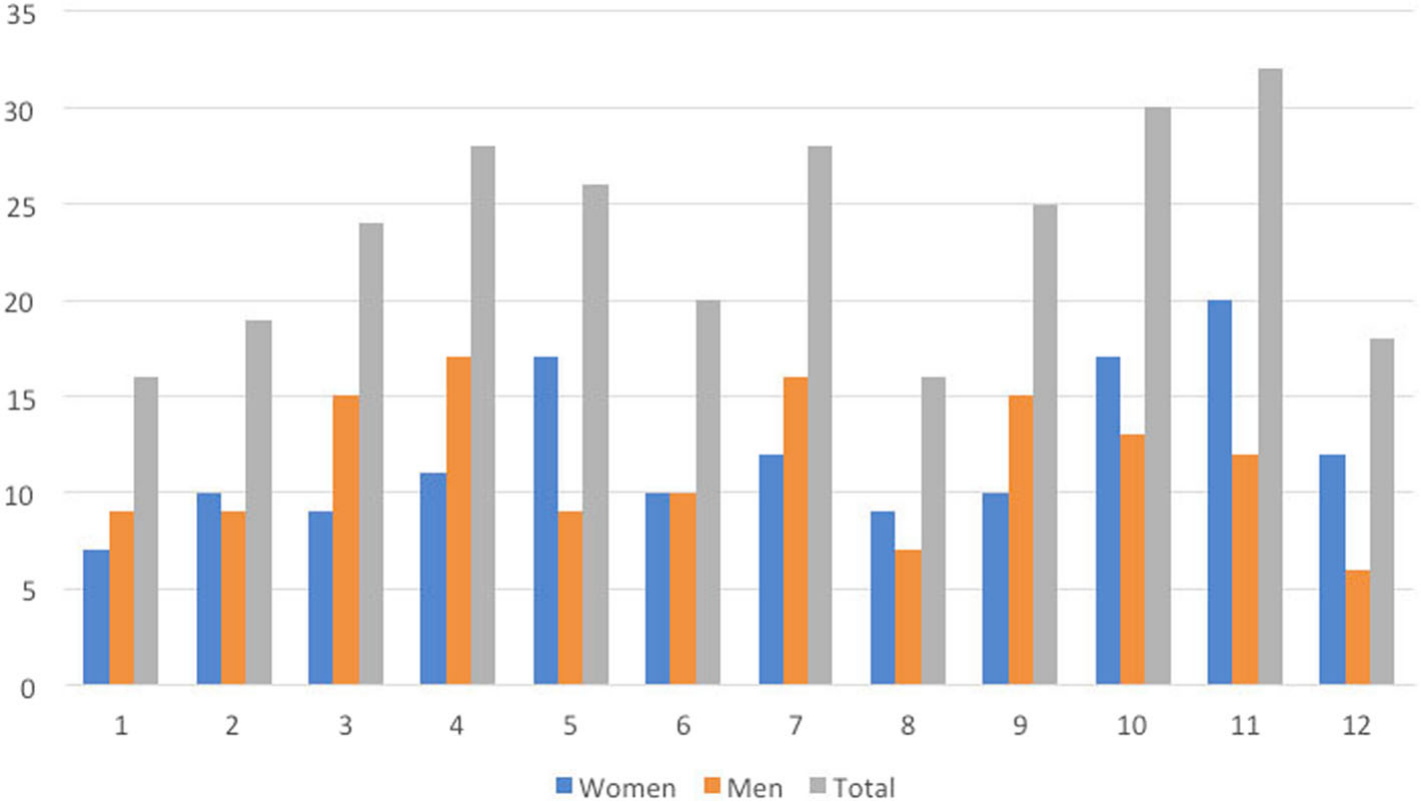
Number of injuries each month. Data missing for 3 women and 1 man

**Fig. 4:**
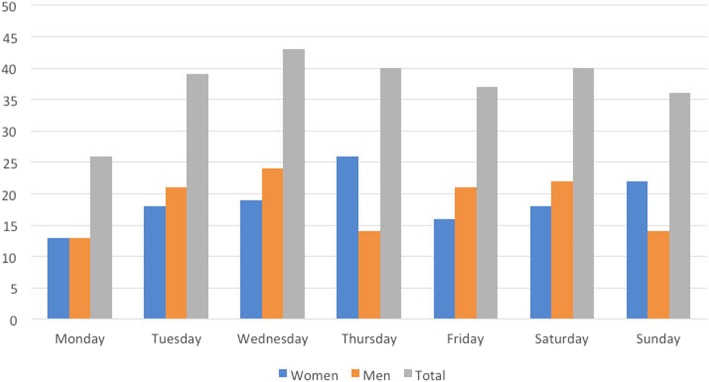
Number of injuries each weekday. Data missing for 13 women and 12 men

Most injuries occurred between 6 and 12 am, with fewer between 0 and 6 am (Fig. 2). Most injuries occurred in November and fewer in January and August (Fig. 3). The highest number of injuries occurred on Wednesdays and lowest on Mondays (Fig. 4).

### Co-morbidity and pharmaceutical drugs

There was no documentation of co-morbidities for 26/286 (9%) patients. Thirty-six (13%) patients were healthy, while the most frequent diagnoses were cardiovascular-metabolic-endocrine diseases and osteoarthritis. One hundred and thirty (45%) patients had hypertension and 96/286 (34%) had cardiovascular diseases. Hypertension (*p* = 0.03), cardiovascular disease (*p* < 0.01), and diabetes mellitus type 2 (*p* < 0.01) were more common among men. Osteoarthritis of the hip and knee (*p* = 0.01), osteoporosis (*p* = 0.01) and thyroid disorders (*p* < 0.01) were more common among women. Other diagnoses, interesting from a hand injury perspective, were dizziness, visually impaired, psychiatric disorders, dementia, and hand surgical disorders. The most frequent diagnoses are shown in Table 2.

There was no documentation of prescribed drugs for 45/286 (16%) patients. The median number of drugs administered was 2 [1–5]. Twenty-two out of two hundred and eighty-six (8%) patients had sustained earlier hand injuries.

### Site, type, injured structure and severity

The most frequent site of injury was the 1st ray for men and the 5th ray for women. Fifty-two (18%) injuries involved multiple sites (Table 3). Fractures were the most common type of injury among women, 97/146 (66%), and wounds among men, 69/141 (49%). The structure most frequently injured was the skin, followed by phalanges and metacarpal bones. Skin and nail-bed wounds were more common among men, while carpal and metacarpal fractures were more common among women. Five patients had only a forearm fracture, but were referred to the department due to suspected scaphoid fracture. Forearm fractures are not routinely followed up at the department, so this number is not representative for forearm fractures in this age group. Forty-two (15%) injuries were to tendons, occurring more commonly among men (*p* < 0.01); 23/287 (8%) injuries were to digital nerves; and 5/287 (2%) to main nerve trunks (Table 3).

**Table 3:** Characteristics for the 287 injuries with a hand injury treated at a department for hand surgery

Injury characteristics	Women *n* = 146	Men *n* = 141	Total *n* = 287	*P*-value
Injured side				
Right	55 (38%)	56 (40%)	111 (39%)	^b^0.78
Left	87 (60%)	79 (56%)	166 (58%)	^b^0.69
Bilateral	4 (3%)	5 (4%)	9 (3%)	–
Missing	0	1 (1%)	1 (0%)	–
Location of injury				
Carpal hand/forearm	27 (18%)	18 (13%)	45 (16%)	^b^0.22
1st ray	18 (12%)	28 (20%)	46 (16%)	^b^0.11
2nd ray	9 (6%)	23 (16%)	32 (11%)	^b^ **0.01**
3rd ray	3 (2%)	6 (4%)	9 (3%)	–
4th ray	14 (10%)	16 (11%)	30 (10%)	^b^0.65
5th ray	48 (33%)	25 (18%)	73 (25%)	^b^ **0.01**
Multiple parts	27 (18%)	25 (18%)	52 (18%)	^b^0.88
Type of injury				
Fracture	97 (66%)	50 (35%)	147 (51%)	^b^ **< 0.01**
Wound	34 (23%)	69 (49%)	103 (36%)	^b^ **< 0.01**
Dislocation/ligament injury	15 (11%)	22 (16%)	37 (13%)	^b^0.21
Injured structures				
Integument				
Skin	33 (23%)	54 (38%)	87 (30%)	^b^ **0.02**
Nail Bed	8 (5%)	23 (16%)	31 (11%)	^b^ **< 0.01**
Skeletal				
Forearm fracture*	4 (3%)	1 (1%)	5 (2%)	–
Carpal fracture	17 (12%)	7 (5%)	24 (8%)	^b^ **0.05**
Metacarpal fracture	40 (27%)	20 (14%)	60 (21%)	^b^ **0.01**
Phalanx fracture	34 (23%)	45 (32%)	79 (28%)	^b^0.16
Dislocation/ligament	13 (9%)	21 (15%)	34 (12%)	^b^0.14
Multiple fractures and/or dislocations	13 (9%)	8 (6%)	21 (7%)	^b^0.31
Tendon				^b^ **< 0.01**
Wrist flexor/extensor tendon	0	5 (4%)	5 (2%)	–
Digital extensor tendon	4 (3%)	11 (8%)	15 (5%)	–
Digital flexor tendon	5 (3%)	11 (8%)	16 (6%)	–
Multiple tendon types	2 (1%)	4 (3%)	6 (2%)	–
Nerve				^b^0.20
Main nerve	2 (1%)	3 (2%)	5 (2%)	–
Digital nerve/radial nerve branch	9 (6%)	14 (10%)	23 (8%)	–
MHISS	12.5 [4–24.5]	20 [9.5–35.5]	16 [6–30]	^a^ **< 0.01**
MHISS groups				
Minor (1–20)	104 (71%)	84 (59%)	188 (65%)	^b^0.22
Moderate (21–50)	33 (23%)	39 (28%)	72 (25%)	^b^0.39
Severe (51–100)	6 (4%)	10 (7%)	16 (6%)	^b^0.29
Major (> 100)	3 (2%)	8 (6%)	11 (4%)	^c^0.12

*Values are presented as number of patients (% of patient group) or median [25th – 75th percentiles]. P-values represent differences between men and women in number of patients affected or median values by the use of* ^*a*^*Mann-Whitney U test for continuous variables and* ^*b*^*Pearson’s Chi-square test or* ^*c*^*Fisher’s exact test (in small sample groups) for categorical variables. Every injured structure is counted for each patient,* i.e. several structures are scored for some patients and consequently sum of column percentage in the category Injured structure is not 100%. *The number of forearm fractures is not representative for the entire population

The median MHISS was significantly higher for men than women (*p* < 0.01). The majority, 260/287 (90%), of the injuries were minor or moderate (Table 3).

### Settings for injury

Documentation concerning settings was missing for 68/287 (24%) injuries. The most common settings for injury, 95/287 (33%), was outdoors, and women sustained more injuries outdoors than men (*p* = 0.05). More men than women (*p* < 0.01) incurred injuries while engaged in carpentry and handling firewood, 34/287 (12%). Twelve out of one hundred and forty-one (9%) men were injured at work and all the injuries were inflicted by power tools or heavy machinery (Table 4).

**Table 4:** Circumstances for the 287 injuries treated at a department for hand surgery

Injury circumstances	Women n = 146	Men n = 141	Total n = 287	*P*-value
Place for injury				
Outdoors	58 (40%)	37 (26%)	95 (33%)	^a^ **0.05**
Home/indoors	34 (23%)	32 (23%)	66 (23%)	^a^0.92
Carpentry/firewood handling	5 (3%)	29 (21%)	34 (12%)	^a^ **< 0.01**
Work	0	12 (9%)	12 (4%)	–
Bus, car motor cycle	7 (5%)	5 (4%)	12 (4%)	^a^0.61
Missing data	42 (29%)	26 (18%)	68 (24%)	^a^0.07
Injury mechanism				
Fall	100 (68%)	53 (38%)	153 (54%)	^a^ **< 0.01**
Cut or saw	16 (11%)	41 (29%)	57 (20%)	^a^ **< 0.01**
Crush or avulsion	10 (7%)	21 (15%)	31 (11%)	^a^ **0.04**
Pull, punch, twist	7 (5%)	14 (10%)	21 (7%)	^a^0.11
Traffic	9 (6%)	6 (4%)	15 (5%)	^a^0.48
Bite	4 (3%)	6 (4%)	10 (3%)	^b^0.49
Specific reason				
Tripping/stumbling/slipping	34 (23%)	29 (21%)	63 (22%)	^a^0.62
Power tool	6 (4%)	30 (21%)	36 (13%)	^a^ **< 0.01**
Animal	11 (8%)	14 (10%)	25 (9%)	^a^0.49
Door/heavy object	8 (5%)	12 (9%)	20 (7%)	^a^0.33
Glass/other sharp object	8 (5%)	8 (6%)	16 (6%)	^a^0.94
Knife/axe/scissors	5 (3%)	9 (6%)	14 (5%)	^a^0.26
Missing data	74 (51%)	39 (28%)	113 (39%)	^a^ **< 0.01**
Other injuries				
Head (wound, contusion, fracture)	15 (10%)	10 (7%)	25 (9%)	^a^0.36
Costal or vertebral fracture/compression	2 (1%)	5 (4%)	7 (2%)	**–**
Lower extremity fracture	4 (3%)	1 (1%)	5 (2%)	**–**

*Values are presented as number of patients (% of patient group) or median [25th – 75th percentiles]. P-values represent difference between men and women in number of patients affected by the use of* ^*a*^ *Pearson’s Chi-square test or* ^*b*^ *Fisher’s exact test (in small sample groups) for the categorical variables*

### Injury mechanism

Falling was more common among women than men (*p* < 0.01) and also constituted (overall) the most common injury mechanism (153/287, 54%). More men than women sustained cut or saw injuries (*p* < 0.01) and crush or avulsion injuries (*p* = 0.04). Tripping/stumbling/slipping was overall the most frequent reason 63/287 (22%) for injury. Accidents with power tools, 36/287 (13%), were more common among men than women (*p* < 0.01). Four patients had cut themselves in suicide attempts. No specified reason was given for 113/287 (39%) injuries, most of which were sustained by women. In 37/287 (13%) injuries, additional body parts, particularly the head, were also injured (Table 4).

### Treatment

More women were treated as out-patients and more men as in-patients (*p* = 0.03). Among the out-patients, men were younger (*p* = 0.03) than women and had a higher MHISS (*p* = 0.01). Most out-patients received conservative treatment; of those receiving operative treatment more were men (*p* = 0.02). The frequency of nurse visits (*p* < 0.01) and use of prophylactic antibiotics (*p* < 0.01) was higher among men, when treated as out-patients (Table 5). Male and female in-patients were similar regarding age and severity of injury and required the same amount of health care (Table 6).

**Table 5:** Treatment data for the 218 out-patients with a hand injury treated at a department for hand surgery

Out-patients				
Treatment	Women *n* = 119	Men *n* = 99	Total *n* = 218	*P*-value
Age	74 [70–79]	71 [68–77]	73 [69–79]	^a^ **0.03**
MHISS	8 [4–20]	12 [6.5–23]	12 [4–20]	^a^ **0.01**
Conservative	105 (88%)	68 (69%)	173 (79%)	^b^0.11
Operative	14 (12%)	31 (31%)	45 (21%)	^b^ **0.02**
Operating time (minutes)	37 [26–66]	30 [15–41.5]	30 [15–45]	^a^0.14
Admitted after treatment	7 (6%)	2 (2%)	9 (4%)	^c^0.19
Out-patient visits				
Hand surgeon	2 [1–2]	2 [1–3]	2 [1–3]	^a^0.14
Nurse	0 [0–0]	0 [0–1.5]	0 [0–1]	^a^ **< 0.01**
Hand therapist	2 [0–3]	1 [0–2]	1 [0–3]	^a^0.08
Antibiotics				
Prophylaxis	15 (13%)	30 (30%)	45 (21%)	^b^ **< 0.01**
Due to infection	3 (3%)	8 (8%)	11 (5%)	^c^ **0.07**
Complications				
Infection	4 (3%)	6 (6%)	10 (5%)	–
Other	1 (1%)	1 (1%)	2 (1%)	–
Re-operations				
1	0	2 (2%)	2 (1%)	–
2 or more	0	0	0	–

*Values are presented as number of patients (% of patient group) or median [25th – 75th percentiles]. P-values represent differences between men and women in number of patients affected or median values by the use of* ^*a*^ *Mann-Whitney U test for continuous variables and* ^*b*^ *Pearson’s Chi-square test or* ^*c*^ *Fisher’s exact test (in small sample groups) for categorical variables*

**Table 6:** Treatment data for the 69 in-patients with a hand injury treated at a department for hand surgery

In-patients in hand surgical ward (other ward)				
Treatment	Women *n* = 27	Men *n* = 42	Total *n* = 69	P-value
Age	73 [70–79]	70.5 [68–73]	71 [68–75]	^a^0.07
MHISS	32 [19–58]	34 [20–74]	32 [20–66]	^b^0.35
Conservative	2 (7%)	(2) (5%)	2 (2) (6%)	^c^0.66
Operative	24 (1) (93%)	36 (4) (95%)	60 (5) (94%)	^b^0.91
Operating time (minutes)	61 [48–90]	64 [40.5–105]	62 [48–98]	^a^0.81
Days in ward	3 [2–4]	2.5 [2–4]	3 [2–4]	^a^0.88
Admitted after treatment	0	2 (5%)	2 (3%)	^c^0.52
Out-patient visits				
Hand surgeon	2 [1–3.5]	2 [1–3]	2 [1–3]	^a^0.57
Nurse	1 [1–3]	2 [1–3]	2 [1–3]	^a^0.37
Hand therapist	3 [0.5–7]	3 [0–6]	3 [0–7]	^a^0.66
Antibiotics				
Prophylaxis	17 (63%)	34 (81%)	51 (74%)	^b^0.40
Due to infection	4 (15%)	4 (10%)	8 (12%)	–
Complications				
Infection	5 (19%)	7 (17%)	12 (17%)	–
Other	2 (7%)	2 (5%)	4 (6%)	–
Re-operations				
1	1 (4%)	3 (7%)	4 (6%)	–
2 or more	2 (7%)	2 (5%)	4 (6%)	–

*Values are presented as number of patients (% of patient group) or median [25th – 75th percentiles]. P-values represent differences between men and women in number of patients affected or median values by the use of* ^***a***^ *Mann-Whitney U test for continuous variables and* ^***b***^ *Pearson’s Chi-square test or* ^***c***^ *Fisher’s exact test (in small sample groups) for categorical variables. For number of patients with conservative and operative treatment, the numbers in the table indicates treatment at a “hand surgical ward” and in “an(other ward)”*

## Discussion

The present study shows that the incidence of hand injuries among the elderly was 21.3/10000 inhabitants and year. Hand injuries occurred to men and women equally, but men were younger than women at the time of injury. Interestingly, the type and severity also differed, with women sustaining fractures and men saw/cut/avulsion/crush injuries; the latter more frequently requiring admission to hospital and surgery and reflected in higher MHISS. Only 13% of the patients were healthy, with hypertension and cardiovascular disease being the most frequent in those who had one or several diseases. Twenty-seven percent of the patients were prescribed ≥5 drugs.

An earlier study, evaluating all age groups in our region, reported an incidence for hand injuries of 70/10000 inhabitants and year, where fractures were the most common injury (49%) and falling was the most common mechanism (41%) [1]. When only the injuries among patients aged 65 years or older in this earlier study were analysed, 55% were found to be fractures and women were more often injured than men. In contrast, the present study shows, when weighted for the number of men and women in this age group in the general population (which was not done in the previous study), that the same proportion of injuries occurred in elderly men and women. A study of 50,272 patients of all age groups in Denmark showed that only 19% of the patients had fractures, but, unlike the present study, it also included contusions, which made up 19% of the injuries [6]. In both of these studies, men sustained the largest number of injuries and the number of wounds was of the same magnitude as in the present study. The studies also showed a reduction in incidence with age. The findings to date show that the elderly do not injure their hands as often as younger patients, but they seem to experience a higher proportion of falls and related fractures.

The most common injury mechanism for fracture, especially in women, was a fall and in 63/153 falls (41%) tripping, stumbling or slipping was the cause. A previous study concerning all kinds of fall-related injuries [12] presents similar findings, where the same causes were also most common (28%). In our study fall-related hand injuries affected twice as many women as men and a similar pattern has been observed in earlier studies [17]. In most cases no specified reasons are given for the rest of the falls. However, some patients fell due to standing on a bus that was braking or were pulled by a dog on a leash. Since fractures from falling constitute the most common injury, their further prevention using proven means, i.e. strength and balance training and vitamin D and calcium supplementation according to a large review of prevention of fall injuries [4], could possibly reduce the number of hand injuries in the elderly.

More men than women sustained injuries from powered wood splitters and circular saws, both of which caused more severe injuries [18–20]. Circular saw injuries occur in both experienced professionals and elderly patients, as recreational work is common also among older adults [18, 19]. Professionals, e.g. carpenters, injure their hands despite years of experience [18]. Traditionally, activities involving various power tools are more common among men, which may explain the present observed difference. Such an argument is also valid for the crush injuries caused by heavy objects, e.g. farming equipment and heavy machinery, since men still engage more often in heavy work than women. Thus, as pointed out in earlier studies [21], the exercise of caution and more safety measures when working with these kinds of equipment cannot be stressed enough if hand injuries among older men are to be reduced.

A majority of the patients had minor or moderate injuries and could be treated as out-patients. Even when the data for out-patients were analysed separately, men had a higher MHISS, required surgery more often than women, required more nurse visits and consumed more prophylactic antibiotics, which is reasonable in light of the more severe and open injuries. More men than women were also admitted to hospital among the patients from the Malmö/Lund regions and from the other regions. However, when the data for the in-patients were analysed separately, men and women did not differ regarding MHISS, operating time, days on the ward and number of out-patient visits. Taken together, these overall data indicate that hand injuries among men are more expensive to treat than those among women.

The observed age difference, with men being younger than women at time of injury, could possibly be explained by the fact that men may still be more active using e.g. power tools during their leisure time or still in a partial professional life in spite of their age. At a higher age one may anticipate that most men decrease their use of hazardous equipment. A consequence of aging, e.g. osteoporosis, especially among women may contribute to observation that women had a higher age at injury. The decrease in overall incidence with age is probably, for several reasons, a result of reduced activity and, thus, less exposure to potentially injurious mechanisms. Osteoporosis was, as expected, more common among women than men. One extensive study on hand fractures concludes that, until retirement, men have a higher relative risk of sustaining hand fractures due to a riskier lifestyle, but after the age of 65 the relative risk is higher for women [22]. That study also found that the most common fractures of the hand are to the metacarpals and phalanges [22]. This is confirmed by the present findings, where women had more fractures than men and the most common damage was to the phalanges and metacarpals. A nationwide study of fall-related injuries from the Netherlands shows that, in comparison with hand/finger fractures, hip fractures were more than four times as common, and wrist fractures 40% more common [13].

Our finding that 58% of the injuries affected the left hand contradicts previous studies in all age groups, where the right hand was more commonly injured, except in cases of work-related hand injuries to right-handed patients [23]. Injuries at work, as well as when engaged in carpentry and handling firewood, were common among men. These latter injuries are comparable to work-related injuries, which might explain the high incidence of left-hand injuries occurring in men. Other studies of severe hand injuries, mainly from saws and machines, also reported an over-representation of left-hand injuries [24, 25]. In older women, the dominant handgrip strength is significantly reduced after the age of 60, and that of the non-dominant handgrip after the age of 50 [26]. The dominant hand is used more often in activities of daily living and hence is stronger and may not fracture as easily as the non-dominant. In older women, reduced handgrip strength is an independent risk factor for osteoporotic fractures [27]. Another study shows that the correlation between non-dominant handgrip strength and frailty is stronger than that between the dominant handgrip strength and frailty [28], indicating that the dominant hand is stronger than the body in general. This might explain the high incidence of left-hand injuries in women. Notably, handedness was poorly documented, making it difficult to draw conclusions from a correlation between handedness and injured side.

The population of older adults in society is increasing as are the numbers of medicines they take. The increase in medication for treatment of various diseases is partly due to more widespread use of cardio-protective medicines and anti-depressants [9]. Patients prescribed more medicines are more likely to have more co-morbidities, more limitations in the activities of daily living and less mental capacity as well as to consume more healthcare than those taking fewer medicines [9]. The influence of side effects of the various medications cannot be excluded, e.g. the risk of orthostatic hypotension in connection with antidepressant treatments. Twenty-seven percent of the present patients took ≥5 drugs, involving the risk of interaction between the different medicines and of side effects. Forty-five per cent of the patients suffered from hypertension and 34% from cardiovascular disease, which is in line with previous findings of co-morbidities in older trauma patients [2]. Considering co-morbidities, medicines and the biological repair processes, repair and reconstruction after a hand injury may be more challenging in elderly patients.

Hand injuries potentially leave the patients with long-term disabilities and functional limitations. Since older people already suffer from a decline in physical and mental capacity they are vulnerable to these consequences and may be in need of additional support after hand injuries. Previous studies have researched the outcome of hand injuries and conclude that a higher severity grade of hand injury correlates with greater costs, longer sick leave and reduced hand function, but that even minor injuries can be debilitating [24, 25, 29]. The outcomes of hand injuries among the elderly have not yet been studied, but the consequences for older patients are likely to affect their ability to engage in daily living and leisure activities. Hence, there is a potential gain from conducting more studies on the topic both in terms of improving the quality of life for the elderly with hand injuries and reducing the costs to society.

There are a number of limitations to this study. The retrospective design precludes any completion of missing data. Some patients with minor hand injuries may have been treated by their general practitioners without referral, despite an established routine for the following up of all hand injuries at our department, meaning that we cannot be sure that all hand injuries were included. As many hand injuries can be treated without knowledge of the patients’ co-morbidities or medicines; thus, documentation of these was sometimes scarce. We believe that it is important for healthcare providers to improve the recording of patient demographics, co-morbidity, handedness etc. with respect to planning of the resources in the health care system. The administrative system for medical records in the primary healthcare and hospital sectors differs and without access to medical records from the primary care sector some co-morbidities and medicines may have been missed. There might be a potential bias when calculating the incidence, which might be underestimated. However, in the present region there is an agreement and specific routines for long with GPs, A&E units and department of Orthopaedics how to refer patients with hand injuries, resulting in that most injuries are treated at the present department.

## Conclusions

In comparison with younger patients, older men and women do not injure their hands as often and the proportion of men and women sustaining injuries is equal. Men are younger at injury, sustain more wounds from hazardous equipment and, thus, have more severe injuries. Women usually fall and fracture their hands. Fall-prevention seems to be most important for reducing injuries among older women, emphasising safety precautions might reduce the injuries among men. A minority of the elderly is healthy, which may complicate treatment. Further studies are needed to examine the outcomes of hand injuries among the elderly.

## Abbreviations

MHISS: Modified Hand Injury Severity Score
